# Exercise Effect on Cerebral Artery Hemodynamic and Morphology in Stroke Patients: A Randomized Trial

**DOI:** 10.1002/cns.70942

**Published:** 2026-05-20

**Authors:** Simon Takadiyi Gunda, Ziman Chen, Jerica Hiu‐Yui Yip, Veronica Tsam‐Kit Ng, Xinyang Han, Jingguo Qu, Xiangyan Chen, Michael Tin‐Cheung Ying, Marco Yiu‐Chung Pang

**Affiliations:** ^1^ Department of Health Technology and Informatics The Hong Kong Polytechnic University Hong Kong SAR China; ^2^ Department of Rehabilitation Sciences The Hong Kong Polytechnic University Hong Kong SAR China

**Keywords:** aerobic exercise training (AET), cerebral artery, hemodynamic, morphology, stroke, ultrasonography

## Abstract

**Aims:**

Stroke is a leading cause of death and disability worldwide and occurs primarily due to impaired cerebrovascular health. Aerobic exercise training (AET) has the potential to improve deconditioned cerebrovascular status in post‐stroke patients, but its utility remains underexplored. This study assessed the effects of AET on cerebral artery morphology and hemodynamics in post‐stroke patients using advanced ultrasonography techniques.

**Methods:**

A randomized controlled trial (RCT) involving post‐stroke patients randomly assigned to either a 12‐week, high‐intensity supervised cycling AET program (3 sessions per week, 30 min per session) or stretching (control) program was conducted. Duplex carotid ultrasound novel applications assessed pre‐ and post‐intervention morphological features (carotid intima‐media thickness (CIMT), arterial stiffness, and 3D features) and hemodynamics (resistive index (RI), pulsatility index (PI)). Transcranial color‐coded Doppler (TCCD) assessed middle cerebral artery (MCA) hemodynamics.

**Results:**

A total of 42 post‐stroke patients, mean age (64.4 ± 7.6 years) were enrolled (cycling AET, *n* = 21 and stretching, *n* = 21). Mixed design ANOVA revealed significant time*exercise group interactions for CIMT, compliance, and distensibility (all *p* < 0.05). Cycling AET significantly improved CIMT (between group mean difference (MD) = −0.04 mm, *p* < 0.043), distensibility (MD = 0.003 (1/kPa)), compliance (MD = 0.11 mm/kPa, all *p* < 0.001). Modest within‐group improvements were observed in internal carotid artery, RI (MD = −0.05, *p* < 0.001), and PI (MD = −0.2, *p* < 0.001). No significant changes observed in MCA hemodynamics.

**Conclusion:**

High‐intensity cycling AET improved cerebral arteries' morphology and modestly enhanced extracranial hemodynamics in post‐stroke patients, without affecting MCA hemodynamics. Further studies are recommended to explore long‐term vascular benefits of AET.

**Trial Registration:**

ClinicalTrials.gov identifier: NCT05706168

## Graphical abstract

This randomized trial assessed the effects of aerobic exercise training (AET) on cerebral artery morphology and hemodynamics in post‐stroke patients as assessed by novel ultrasonography techniques. High‐intensity cycling AET improved cerebral artery morphology and arterial stiffness, offering a non‐invasive intervention for cerebrovascular remodeling with the potential to mitigate future stroke recurrence.

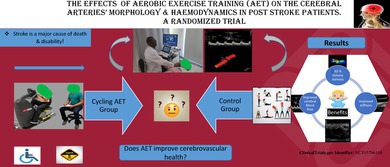

## 1 Introduction

Stroke remains a leading cause of death and long‐term disability worldwide [1] and occurs primarily due to impaired cerebro‐vasculature among other causes [2, 3, 4]. It is characterized by manifestations of neurological deficits in cognitive and motor domains [5, 6]. To improve quality of life and survival, robust management strategies are required. As the cerebrovascular system plays a crucial role in stroke etiology [2], and is deconditioned following a stroke, it can be posited that treatment strategies targeting to enhance cerebral arteries' structural and functional features could significantly influence post‐stroke recovery and mitigate against future stroke recurrences. Current treatments, including antiplatelet medication such as (aspirin and clopidogrel) and endovascular treatments, are not without challenges. Their narrow therapeutic windows often result in stroke progression to chronic phase [7, 8], while their invasiveness and failure rates of up to 20% further reduce efficacy [9]. It is thus of growing demand for effective, well tolerated, non‐invasive post‐stroke alternative treatment strategies.

Aerobic exercise training (AET), a non‐invasive and non‐pharmacological intervention, has the potential to restore deconditioned cerebrovascular status via the principal mechanism of endothelium cell stimulation by shear stress to produce increased nitric oxide (NO), a known mediator of endothelial function [10, 11]. Cycling is a common type of AET, with potential application in post‐stroke patients due to its safety. Although the beneficial effects of AET on systemic vascular health are established in the general population [12, 13, 14, 15], limited studies have explored AET's impact on cerebral arteries' morphological and haemodynamic features, particularly in chronic post‐stroke patients [16, 17]. Existing studies have also reported contradictory findings [18, 19]. Besides, only a few cerebrovascular function indicators were assessed, mainly (1) cerebral vasomotor reactivity (cVMR) and (2) mean flow velocity (MFV) of the middle cerebral arteries (MCAs) based on non‐imaging transcranial Doppler ultrasound (TCD). However, over recent years, medical imaging modalities, particularly ultrasonography, have evolved with the emergence of novel duplex carotid ultrasonography (DCUS) applications not limited to three‐dimensional (3D) carotid ultrasound [20, 21, 22, 23], and automated arterial stiffness analysis [24]. Additionally, transcranial color‐coded Doppler (TCCD) ultrasound, an advancement to non‐imaging TCD in assessing intracranial cerebral arteries' hemodynamics, is now readily available [25, 26]. These new applications can provide non‐invasive, accurate, and reliable assessments of cerebrovascular health, thus a better assessment of the efficacy of rehabilitation strategies.

Considering this background, this study assessed the effects of cycling AET on cerebral arteries' morphological and hemodynamic features in post‐stroke patients using multi‐parametric advanced ultrasonography techniques. We hypothesized that cycling AET improve cerebro‐vascular features.

## 2 Methods

### 2.1 Standard Protocol Approvals, Registrations, and Patient Consents

This was a single center, parallel group, randomized controlled trial (RCT) approved by the Institutional Review Board (IRB) of The Hong Kong Polytechnic University (HSEARS20220714001) and registered according to WHO recommendations for conducting clinical trials. All participants provided written informed consent prior to undertaking the study.

### 2.2 Study Population

Community dwelling post‐stroke patients were prospectively and consecutively recruited between 10 February 2023 and 31 August 2024. The inclusion criteria were as follows: (1) post‐stroke adults' patients of Chinese origin, aged 50 years or older, (2) patients in chronic phase (time of stroke onset > 6 months) (3) those with mild to moderate disability to undertake cycling AET and stretching exercises and (4) those not participating in any structured AET program. Exclusion criteria were those: (1) non‐consenting, (2) participating in any structured AET program, (3) severely non‐ ambulatory, and (4) allergic to ultrasound gel. We restricted inclusion to patients aged > 50 years to study a more clinically homogeneous population and reduce age‐related variability in baseline physical capacity, and exercise response. Those meeting all inclusion criteria were enrolled and randomly assigned into either a supervised cycling AET or supervised stretching (control) group. Randomization was conducted based on block randomization with no randomization restriction at 1:1 ratio as participants were enrolled in batches and not all at once. This was performed using an online software available at https://www.graphpad.com/quickcalcs/randomize1.cfm. One research team member (J.H.Y.) enrolled participants and generated the allocation sequence, while another team member (V.T.N.) assigned participants to intervention and control groups and participants were blinded of group allocation. Allocation concealment was maintained by ensuring that the group assignment was not known prior to participant baseline assessment. The assessor (S.T.G.) who performed experiments to obtain outcome measures was not involved in the randomization and was blinded to final group allocation. Furthermore, the 12‐week interval between baseline and post‐intervention assessments was sufficiently long to minimize the risk of recall bias.

Changes were made to the initial protocol, which had suggested 3 treatment arms due to treadmill training safety related issues as a safety harness could not be timely secured and these changes were made prior to any participant screening. The CONSORT checklist was used in writing our report [27].

### 2.3 Sample Size Calculation

An a priori power analysis was conducted using G*Power software for a two‐tailed *t*‐test (Difference between two independent means). Based on calculated effect size, Cohen *d* = 0.84 [28], *α* = 0.05, and statistical power = 80%, the current study targeted a sample size of 48 participants (~24 in each group‐cycling AET and stretching‐control).

### 2.4 Data Collection Methods and Tools

Patients' demographics, medical history, and cerebral arteries' hemodynamic and morphological outcome features were collected at our institutional laboratories and coded for anonymity.

### 2.5 Patient's Demographic, and Medical History

Demographics and medical history data were collected via questionnaires. Blood pressure (BP) and heart rate (HR) were measured with an Omron automatic monitor (HEM‐8712, Omron healthcare manufacturing Vietnam Co. Ltd. Vietnam). Participants were classified as hypertensive when systolic blood pressure (BPs) was ≥ 140 mmHg, diastolic blood pressure (BPd) ≥ 90 mmHg, or if they had a clinical diagnosis of hypertension and were receiving treatment, regardless of measured BP values [29].

### 2.6 Cerebral Arteries' Hemodynamic and Morphological Features Assessment

Cerebral arteries' hemodynamic and morphological features were assessed at two time points: (1) baseline (before cycling AET or stretching program) and (2) after 12 weeks of cycling AET or stretching program. A sole experienced sonographer performed DCUS and TCCD ultrasound examinations using a Samsung RS85 ultrasound machine (Samsung Medison Co. Ltd., Republic of Korea), equipped with (1) high frequency linear array transducer (2–14 MHz), (2) 3D linear volumetric transducer (3–14 MHz), and (3) 1–5 MHz phased array. The DCUS protocols involved scanning bilateral carotid arteries with the patient lying supine. Longitudinal and transverse scans of common carotid arteries (CCA) and internal carotid arteries (ICA) were performed in grayscale mode, followed by spectral Doppler and 3D arterial analysis. Morphological features assessed included: (1) carotid intima‐media thickness (CIMT), (2) carotid arterial stiffness indices—β‐stiffness index (CAS β), pulse wave velocity (CAS PWV), elastic modulus (CAS kPa), carotid compliance (CAS CC), and distensibility coefficient (CAS DC), (3) 3D arterial features—carotid plaque volume (CPV), carotid vessel wall volume (CVWV), and 3D carotid lumen volume stenosis (%) (CLVS). Carotid arteries and MCAs' hemodynamic parameters—peak systolic velocity (PSV), end diastolic velocity (EDV), MFV, resistive index (RI), and pulsatility index (PI) were also assessed.

To ensure validity and reliability of measurements, ultrasound scan settings were optimized and standardized for pre‐ and post‐intervention measurements, and an average for 3 measurements was used. Additionally, intra‐rater reliability analysis was performed using ICC (1, k). Below are ultrasound protocols for each specific parameter.

### 2.7 Carotid Intima‐Media Thickness (CIMT)

Using an automated arterial analysis quantification program, CIMT was measured as distance between lumen‐intima boundary (LIB) and media‐adventitia boundary (MAB) in a 1‐cm segment of distal CCA far wall, starting at the inferior margin of carotid bulb. The region of interest (ROI) was consistently used across all assessments.

### 2.8 Carotid Arterial Stiffness (CAS)

CAS indices were evaluated by contouring two parallel lines over the far and near wall of distal CCA using a semi‐automated arterial analysis software. The mathematical representation of CAS parameters was informed by Yuan et al. [24]. Higher CAS β, PWV, and kPa values represented stiffer arteries, whereas higher CAS CC and DC values suggested decreased stiffness.

### 2.9 Three‐Dimensional Carotid Ultrasound

The single‐point acquisition protocol was used to acquire 3D datasets, including (1) carotid lumen volume stenosis (%) (CLVS), (2) CVWV, and (3) CPV. The technique involved holding the 3D transducer still at a single fixed position corresponding to the inferior margin of the carotid bulb. The transducer automatically scanned through the artery segment in a 0.5 mm slice thickness and a 30 degrees sweep angle. The image quality optimization strategy was similar to gray scale imaging, with however overall gain lowered by about 10% to avoid reverberation artifact being contoured as plaque region.

### 2.10 Transcranial Color‐Coded Doppler (TCCD) Ultrasonography

The TCCD protocol involved scanning the MCAs through transtemporal windows using angle correction (cTCCD) technique as previously described [26]. All MCAs hemodynamic parameters were measured with participants in a resting state. Furthermore, pre and post TCCD imaging depths were standardized for each participant to mitigate potential influence of the interrogation depth on MCA haemodynamic measurements as highlighted in our previous study [26].

### 2.11 Cycling AET Equipment

A cycle ergometer, ISO1000R Isokinetic Recumbent Bike (Scifit, USA), offering forward resistance in conjunction with a Polar T31‐coded chest strap heart rate monitor was used to deliver 36 sessions of aerobic exercises over a 12‐week period. The heart rate program freely available on the bike was selected and the chest strap linked to the recumbent bike via a Bluetooth connection was used to continuously monitor HR during exercise.

### 2.12 Exercise Prescription

We targeted an exercise dosage consisting of (1) session duration = 30 min, (2) frequency = 3 times/week, (3) high intensity (HIT) = 60%–84% heart rate reserve (HHR), (4) type = cycling ergometry, and (5) overall program duration = 12 weeks. The exercise intensity was prescribed based on the % HRR method as it considers the resting HR unlike maximum heart rate (HR_max_) method. The target HR was calculated as follows: target HR = [(age‐predicted HR_max_ − resting HR) × % intensity desired] + resting HR (Karvonen formula). Before attaining targeted high intensity training mode, an initial two‐week acclimatization period at moderate intensity (40%–59% HRR) was adopted during the first 6 sessions. Thereafter a high‐intensity protocol was adopted for remaining sessions. The 30‐min session duration consisted of a 2‐min warm‐up cycling at 25 watts power, followed by 23 min of continuous cycling within target heart rate zone, and lastly 5 min unloaded, cool down (zero watts). The cool down time of 5 min was inclusive of between continuous cycling recovery periods, where applicable. During the continuous cycling period, power output was automatically adjusted until the target HR zone was reached and participants were tasked to cycle within the target heart rate zone at self‐selected cadence. A continuous 23‐min HIT session was chosen to provide a simplified, standardized and clinically feasible high‐intensity stimulus while minimizing the complexity and variability associated with prescribing repeated work–recovery intervals.

### 2.13 Stretching Exercises Training

Controls were engaged in simple, non‐aerobic stretching exercises involving dynamic and static stretches of upper and lower limbs, conducted over a similar duration to cycling AET. The aim was to maintain physical activity levels without introducing the cardiovascular demands of aerobic exercise, allowing for a clear comparison with the cycling AET group.

During entire training sessions, close monitoring of HR for any cardiovascular intolerance and checking signs of fatigue was done. As a safety precautionary measure, the exercise session would be terminated when (1) HR increased above high intensity exercise zone or failed to restore back to warm‐up values during recovery, (2) observed signs of severe fatigue, excessive sweating or if the participant indicated he/she couldn't continue.

Furthermore, to allow for protocol adherence tracking, all exercise sessions for both groups were carried out at our institutional laboratories and each session was closely monitored and recorded.

### 2.14 Data Analysis

The intention to treat analysis protocol was adopted and statistical analyses were performed using IBM SPSS version 26 at end of trial, with no interim analyses. Continuous data was expressed as mean ± SD, whereas categorical data was presented as frequencies (%). Kolmogorov Smirnov and Levene's homogeneity of variance tests checked data for normality and homogeneity of variance, respectively. For each cerebral artery morphological feature, the primary analysis was two‐way mixed ANOVA (unadjusted) used to test the time*group interaction effect, whereas hemodynamic features were assessed initially using the two‐way mixed ANCOVA (adjusted for baseline characteristics). These were followed by univariate analyses. In the SPSS analysis, the independent variables were time (within‐subject factor) and exercise group (between‐subject factor). Pre‐to‐post intervention changes within each exercise group were assessed using paired *t*‐tests or Wilcoxon signed‐rank tests, as appropriate. The absolute difference between post and pre‐exercise values within each group represented the within‐group mean difference (MD). The between‐group differences in MDs for morphological and hemodynamic parameters were assessed using one‐way ANOVA (unadjusted) and one‐way ANCOVA (adjusted for covariates), respectively.

The between‐group MD was further expressed as a standardized mean difference (SMD), representing the overall effect size, calculated as Cohen's *d* = overall group MD/pooled SD, given equal sample sizes in the two groups. Effect sizes were computed using a free online calculator available at https://www.socscistatistics.com/effectsize/default3.aspx, and categorized as small (0.2–0.5), medium (0.5–0.8) or large (≥ 0.8) as informed by studies [30, 31].

Improvement was defined as a significant decrease in CIMT, CAS β, PWV, kPa, CPV, CVWV, CLVS (%), RI, and PI, or a significant increase in CAS CC and DC. Statistical significance was set at *p* < 0.05. Between‐group differences in percentage of those showing improvement were computed using Chi‐squared test (or Fisher's Exact test if criteria for Chi‐squared were not fulfilled). The Protocol and statistical analysis plans are available at https://clinicaltrials.gov/study/NCT05706168.

## 3 Results

**FIGURE 1:**
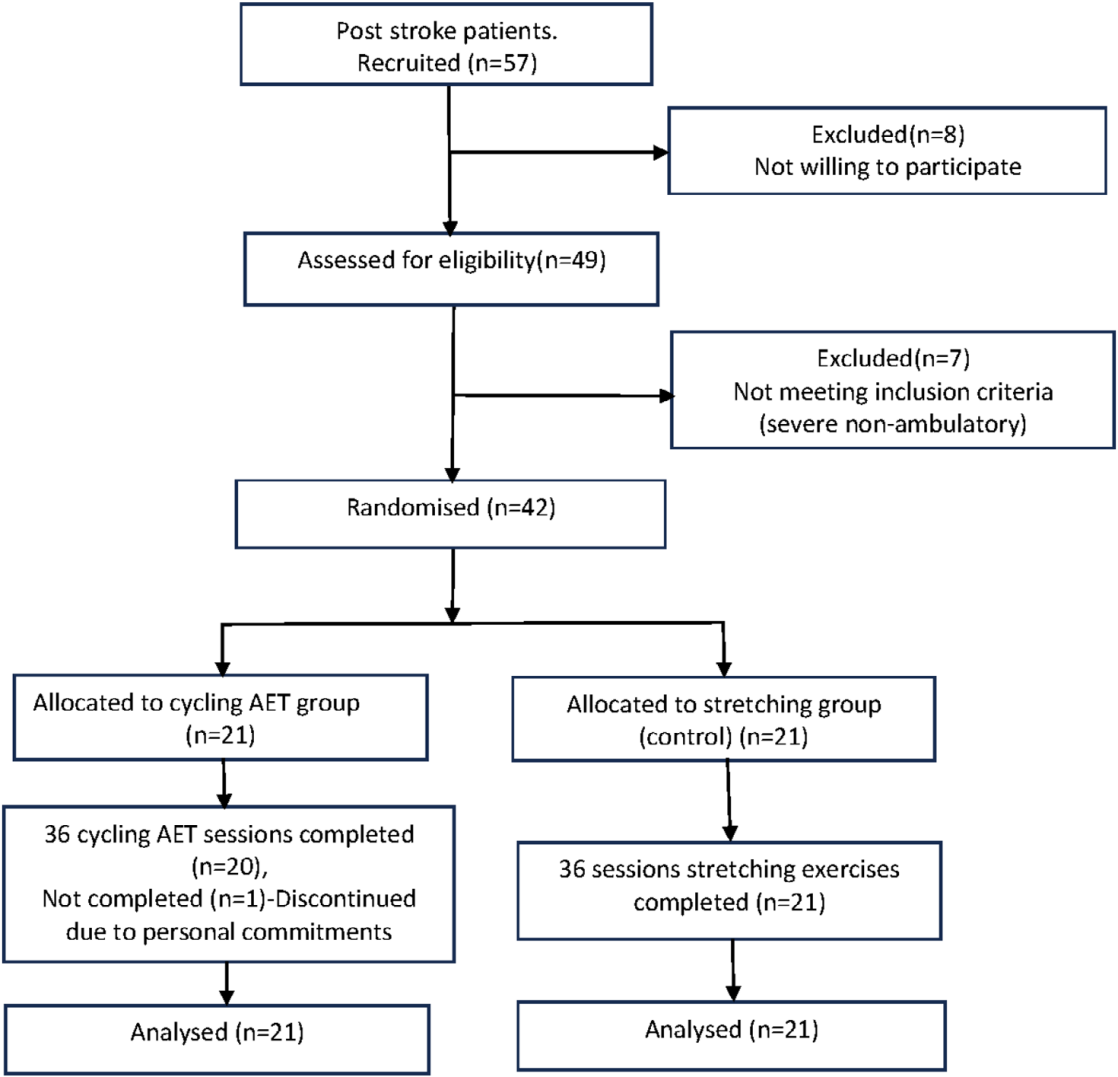
CONSORT flow diagram.

A total of 57 post‐stroke patients with stroke onset time > 6 months were recruited into the study. Eight declined to participate, and seven were excluded for not meeting the inclusion criteria (rejection reason being completely non‐ambulatory to undergo the exercise program). The remaining 42 participants were randomized to the cycling AET group (*n* = 21) or the stretching (control) group (*n* = 21). All participants completed 36 exercise sessions as per protocol, except one cycling AET participant who completed only 11 sessions due to personal commitments (protocol adherence rate = 98%). Based on intention to treat analysis, data for all 42 randomized patients was considered for analysis. The study participants selection process is shown in (Figure 1).

### 3.1 Demographic Characteristics and Clinical History Data for the Post‐Stroke Participants

The baseline demographic and clinical characteristics of study participants in the two groups—cycling AET (interventional) and stretching (control) groups are presented in (Table 1). Baseline demographic and morphological characteristics were homogeneous between groups (all *p* > 0.05) allowing for a fair between group comparison. The mean age of all participants was 64.4 ± 7.6 years, and corresponding cycling AET and stretching groups' participants mean age was 62.1 ± 6.9 years, and 66.7 ± 7.7 years, respectively (*p* = 0.051). Although only 11 (26%) of subjects had systolic blood pressure above or equal to 140 mmHg indicative of hypertension, a total of 31 (73.8%) were classified as hypertensive. Furthermore, the proportion of participants taking blood pressure‐lowering medication did not differ significantly between the two groups (8 (38.1%) in the cycling AET group vs. 12 (57.1%) in the control group, *p* = 0.22). We observed a greater proportion of patients 27 (64%) with Ischemic stroke compared to haemorrhagic stroke. However, proportions of stroke type were not significantly different across the two exercise groups (*p* = 0.334). Additionally, all categorical demographic characteristics, values did not significantly differ between groups (all Chi‐squared *p* > 0.05).

**TABLE 1:** Baseline demographics and clinical characteristics of study participants.

Baseline characteristic	Groups			Between group differences
	All (*n* = 42)	Cycling AET (*n* = 21)	Stretching control (*n* = 21)	*p*
Gender (male/female)	22/20 (52/48)	12/9	10/11	0.537
Age (years)	64.4 ± 7.6	62.14 ± 6.9	66.7 ± 7.7	0.051
Weight	63.2 ± 12.2	66.3 ± 13.9	60.1 ± 9.5	0.098
Height (cm)	161 ± 8	163 ± 8.6	158.9 ± 8.9	0.133
BMI (kg/m^2^)	24.3 ± 3.8	24.97 ± 4.6	23.57 ± 2.7	0.241
BPs (mmHg)	126 ± 15.9	125 ± 17	127 ± 14	0.662
BPd (mmHg)	78 ± 8.7	77 ± 8	79 ± 9	0.371
HR (bpm)	74 ± 11.2	74.1 ± 11.9	73.3 ± 10.7	0.819
Hypertension/No (%)	31/11 (74/26)	14/7	17/4	0.292
Hyperlipidemia/No (%)	26/16 (62/38)	12/9	14/7	0.525
Diabetes mellitus/No (%)	12/30 (29/71)	7/14	5/16	0.495
Type of stroke: Ischemic/hemorrhagic (%)^a^	27/15 (64/36)	15/6	12/9	0.334
Stroke Onset time (years)		7.6 ± 9.1	4.9 ± 4.5	0.222
Age at stroke onset		54.5 ± 11.7	61.8 ± 7.5	0.021
Smoking history/No	1/41 (2/98)	0/21	1/20	0.311

Abbreviations: BMI, body mass index (kg/m^2^); BPd, Diastolic Blood pressure; bpm, Beats per minute; BPs, Systolic Blood pressure; HR, Heartrate; MD, mean difference.

^a^ Type of stroke‐Ischemic/hemorrhagic, continuous data is expressed as mean ± SD and independent *t*‐test *p*‐values are displayed, categorical data is expressed as frequencies and percentages in parentheses (%), *p*‐values, *χ* ^2^‐Chi squared test.

### 3.2 Carotid Arteries' Morphological Features (Pre and Post Interventional) for Cycling AET and Stretching (Control) Groups

**FIGURE 2:**
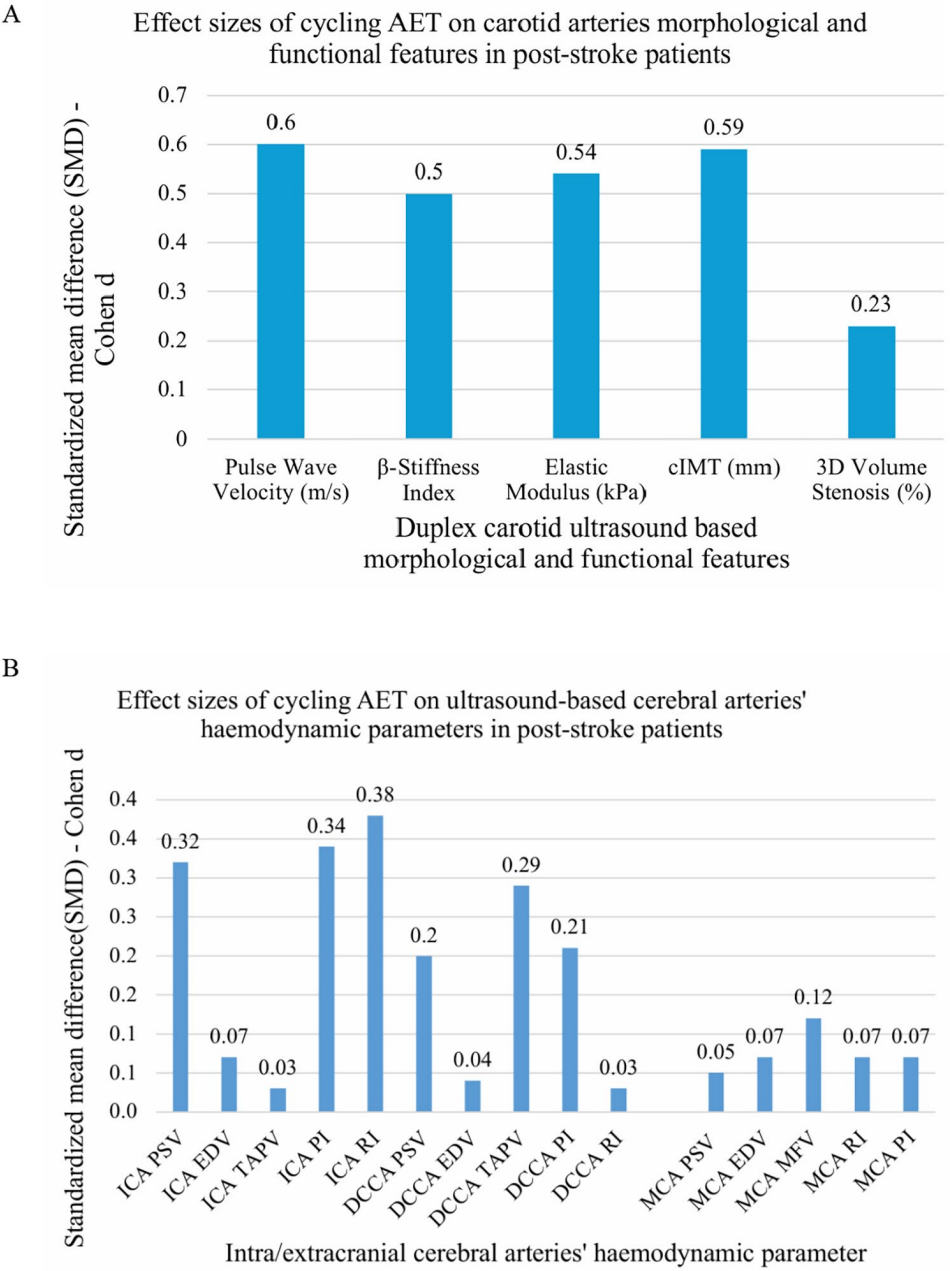
Histogram showing standardized mean differences between cycling AET and stretching (control) group on cerebral arteries characteristics. (A) Morphological features and (B) hemodynamic parameters.

Pre and post carotid arteries' morphological and arterial stiffness parameters of cycling AET and stretching (control) groups and their mean differences are shown in (Table 2) and Figures S1–S3. Baseline comparisons of the morphological features demonstrated between groups homogeneity across all evaluated parameters (all *p*‐values > 0.05). The mixed design ANOVA showed significant main effect of time (*F* = 26.83, *p* < 0.001) and time*exercise group interactions (*F* = 4.252, *p* = 0.042) on CIMT indicating that changes in CIMT over‐time varied significantly between exercise groups. The intervention group showed a mean reduction in CIMT from 0.85 to 0.78 (change = −0.07), while the control group changed from 0.82 to 0.79 (change = −0.03) (net cycling AET effect on CIMT was −0.04, *p* = 0.043*). Additionally, there was a significant main effect of time across all stiffness indices and 3D features (*p* < 0.05). However, time*group interaction was only significant for CAS CC (*F* = 4.584, *p* = 0.035), and CAS DC (*F* = 4.138, *p* = 0.045). Furthermore, significant improvements in all carotid artery morphological features in cycling AET group, including reductions in CIMT, arterial stiffness (CAS β, PWV, kPa), CPV, CVWV, and 3D‐CLVS (%) as well as increases in CAS CC, CAS DC (all *p* < 0.05) were observed in cycling AET. Contrarily, only CIMT and 3D‐CLVS (%), improved in stretching group with less pronounced changes compared to cycling group. Between‐group comparisons revealed significant differences in CIMT, CAS CC, and CAS DC (all *p* < 0.05). The overall effect size of cycling AET on carotid arteries' morphological and arterial stiffness features based on Cohen *d*, standardized mean differences (SMD) are shown in (Figure 2A). In all assessed features a medium effect size was noted except in 3D‐based carotid lumen volume stenosis where the effect size was small, Cohen *d* = 0.23 (Figure 2B).

**TABLE 2:** Carotid arteries' morphological features for the cycling AET (interventional) and stretching (control) groups and mean differences (MD).

Carotid arteries morphological outcomes	Exercise groups								Between group comparisons		
	Cycling AET—Interventional (*n* = 42)				Stretching—Control (*n* = 42)				Btwn group MD		Btwn group baseline, *p*
	Pre‐cycle	Post‐cycle	Cycle MD, 95% CI	Within cycle, *p*	Pre‐stretch	Post‐stretch	Stretch MD, 95% CI	Within stretch, *p*	MD, 95% CI	*p*	*p*
CIMT (mm)	0.85 ± 0.25	0.78 ± 0.2	−0.07 (−0.1, −0.04)	< 0.001*	0.82 ± 0.18	0.79 ± 0.17	−0.03 (−0.05, −0.007)	0.012*	−0.04 (−0.08, 0.001)	0.043*	0.497
CAS PWV (m/s)	8.02 ± 4.3	6.17 ± 1.7	−1.85 (−2.9, −0.8)	0.001*	7.39 ± 4.0	6.57 ± 1.86	−0.82 (−2.1, 0.5)	0.213	1.02 (−0.63, 2.68)	0.221	0.490
CAS β	17.6 ± 28.7	8.6 ± 6.9	−9.0 (−16.2, −1.7)	0.017*	14.5 ± 31.0	9.5 ± 8.8	−5.0 (−15.1, 5.1)	0.325	−3.95 (−16.22, 8.33)	0.524	0.641
CAS kPa	234.4 ± 389	114 ± 91.3	−120 (−219, −19.8)	0.02*	186.4 ± 346	125 ± 93.5	−61 (−173, 51)	0.277^†^	−59.0 (−206.5, 89.1)	0.431	0.552
CAS CC (mm/kPa)	0.48 ± 0.3	0.62 ± 0.3	0.14 (0.06, 0.23)	0.001*	0.52 ± 0.3	0.55 ± 0.2	0.03 (−0.04, 0.1)	0.393	0.11 (0.008, 0.221)	0.035*	0.514
CAS DC (1/kPa)	0.009 ± 0.005	0.01 ± 0.006	0.003 (0.001, 0.005)	0.001*	0.01 ± 0.007	0.01 ± 0.004	0 (−0.002, 0.002)	0.919	0.003 (0, 0.006)	0.045*	0.484
3D CLVS (%)	5.9 ± 5.1	3.5 ± 3.5	−2.4 (−3.5, −1.3)	< 0.001*	5.36 ± 4.1	4.16 ± 3.2	−1.2 (−2.3, −0.1)	0.032*	−1.2 (−2.7, 0.4)	0.129	0.590
3D CPV	148.7 ± 142	88.7 ± 93.1	−60.0 (−95.2, −24.7)	0.001*	129.3 ± 103	103 ± 91	−26.3 (−53.5, 0.9)	0.058	−33.7 (−77.6, 10.2)	0.130	0.478
3D CWV	764.9 ± 185	709.6 ± 132	−55 (−105, −5.2)	0.031*	723.8 ± 180	715.4 ± 145	−8.4 (−58.8, 420)	0.738	−46.9 (−116, 23.1)	0.186	0.308

Abbreviations: 95% CI, confidence interval; Btwn, between; CAS CC (mm/kPa), carotid compliance coefficient; CAS DC (1/kPa), distensibility coefficient; CAS kPa, Elastic modulus; CAS PWV (m/s), carotid arteries pulse wave velocity; CAS β, Beta stiffness index; CIMT (mm), mean carotid intima media thickness of 1 cm long ROI in the distal common carotid artery; CLVS (%), carotid lumen volume stenosis (%); CPV, carotid plaque volume (mm^3^); CWV, carotid wall volume (mm^3^); MD, mean difference (post minus pre) values; *n*, number of vessels (left and right side) from 21 subjects in each group.

* *p* < 0.05 significance level.

^†^ *p*, nonparametric.

**FIGURE 3:**
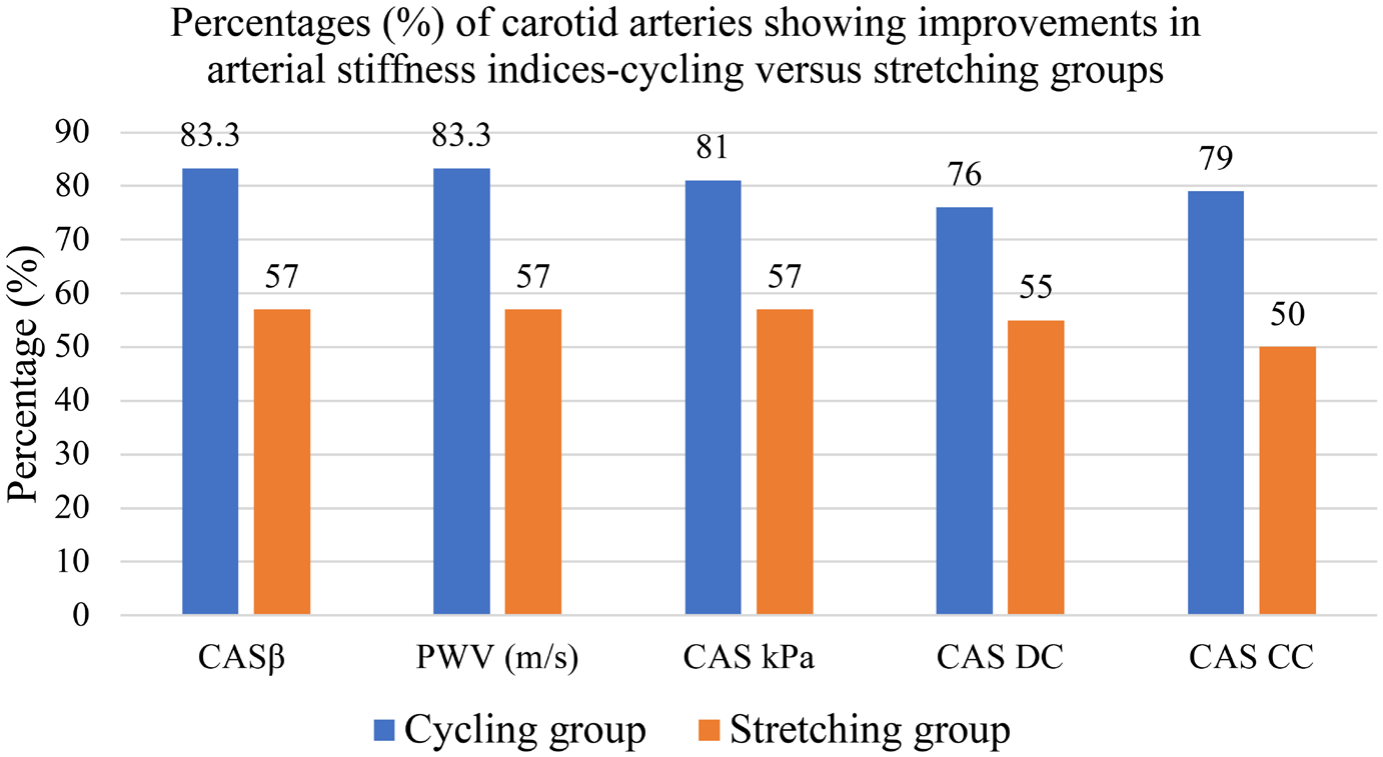
Histogram showing percentages (%) of carotid arteries with improvements in arterial stiffness indices for the cycling and stretching groups.

Furthermore, Chi‐squared analysis of carotid arteries exhibiting improvements in arterial stiffness revealed that the cycling AET group had significantly greater percentages (%) of participants with arterial stiffness improvements compared to the stretching group (all indices, *p* < 0.05), except distensibility coefficient, which showed comparable proportions between the two groups (cycling = 32 (76%) versus stretching = 23 (54.8%), *p* = 0.083). Decreases in PWV and CAS β stiffness indices were observed in 35 (83%) of the cycling group's vessels, whilst the elastic modulus decreased in 34 (81%) of vessels. In the stretching group, corresponding percentages of vessels in which PWV, CAS β, and elastic modulus decreased were each 24 (57%) and lower than those in the cycling group. Additionally, a greater proportion of cycling AET participants showed increased carotid compliance, 33 (79%) compared to the stretching group's 21 (50%), *p* = 0.012 (Figure 3). The between‐group difference *p* values were respectively (PWV = 0.009, CAS β = 0.009 and CAS kPa = 0.018, CAS DC = 0.083 and CAS CC = 0.012).

### 3.3 Cerebral Arteries' Hemodynamic Parameters (Pre and Post) for Cycling AET and Stretching (Control) Groups and Mean Differences (MD)

The pre and post cerebral arteries' hemodynamic parameters for cycling AET and stretching groups and their respective MDs are shown in (Table 3). Significant reductions in measures of RI and PI were observed in both DCCA and ICA segments for cycling AET group whereas in stretching group such changes were only observed in ICA segment. The MD (95% CI) for cycling AET, ICA PI was −0.2 (−0.3; −0.09, *p* < 0.001), whereas corresponding values for stretching were −0.1 (−0.18; −0.02, *p* = 0.018). The ICA RI MD (95% CI) for cycling AET and stretching groups were −0.05 (−0.08; −0.3, *p* < 0.001) and −0.03 (−0.05; −0.006, *p* = 0.013) respectively. Furthermore, the DCCA EDV MD (95% CI) for cycling AET and stretching groups were 1.6 (0.58; 2.7), *p* = 0.003* and 1.5 (0.43; 2.55), *p* = 0.007*. Although significant within‐group changes were observed in some hemodynamic parameters, the between‐group comparison of the MDs showed no statistically significant difference across all hemodynamic parameters even after adjustments of covariates (baseline ICA PSV, DCCA PSV and TAPV). In the present study both unadjusted and adjusted results for hemodynamic parameters are reported. Although the notion of baseline adjustment is still debated [32], agencies including European Medicines Agency and US Food and Drug Administration have recommended baseline adjustments as it is deemed to improve statistical efficiency [33].

**TABLE 3:** Cerebral arteries' haemodynamic features for the cycling AET (interventional) and stretching (control) groups and the mean differences (MD).

Hemodynamic parameter	Groups								Between group comparisons				
	Cycling AET—Interventional (*n* = 42)				Stretching—Control (*n* = 42)				Btwn group MD^a^—no baseline adjustments		Btwn group MD^b^—baseline adjustments		Btwn group baseline, *p*
	Pre‐cycle	Post‐cycle	Cycle MD, 95% CI	Within‐cycle, *p*	Pre‐stretch	Post‐stretch	Stretch MD, 95% CI	Within stretch, *p*	MD^a^, 95% CI	*p*	MD^b^, 95% CI	*p*	*p*
**Carotid arteries**													
ICA PSV (cm/s)	67.7 ± 25.6	63.0 ± 18.0	−4.7 (−11.2;1.7)	0.148	57.9 ± 14.7	58.8 ± 13.4	0.95 (−3.4;5.3)	0.660	−5.7 (−13.4, 2.0)	0.144	1.0 (−5.4, 7.4)	0.758	0.034*
ICA EDV (cm/s)	21.8 ± 7.9	23.6 ± 7.8	1.7 (−0.4;3.8)	0.106	19.7 ± 8.0	21.2 ± 7.2	1.6 (−0.4; 3.5)	0.120	0.16 (−2.7, 3.0)	0.914	2.2 (−0.69, 5.03)	0.134	0.214
ICA PI	1.3 ± 0.4	1.1 ± 0.3	−0.2 (−0.3; −0.09)	< 0.001*	1.26 ± 0.48	1.16 ± 0.4	−0.1 (−0.18; −0.02)	0.018*	−0.1 (−0.23, 0.03)	0.125	−0.1 (−0.26, 0.04)	0.134	0.869
ICA RI	0.68 ± 0.1	0.63 ± 0.09	−0.05 (−0.08; −0.3)	< 0.001*	0.67 ± 0.09	0.64 ± 0.08	−0.03 (−0.05; −0.006)	0.013*	−0.02 (−0.06, 0.01)	0.099	−0.03 (−0.07, 0.01)	0.107	0.435
DCCA PSV (cm/s)	62.4 ± 16.3	61.1 ± 18.1	−1.3 (−5.04; 2.4)	0.489	55.4 ± 12.1	56.3 ± 12.7	0.9 (−2.29; 4.0)	0.559	−2.2 (−6.98, 2.59)	0.364	−0.75 (−5.90, 4.40)	0.772	0.027*
DCCA EDV (cm/s)	17.0 ± 5.2	18.6 ± 5.2	1.6 (0.58; 2.7)	0.003*	15.3 ± 3.8	16.8 ± 4.4	1.5 (0.43;2.55)	0.007*	0.1 (−1.34, 1.62)	0.847	0.4 (−1.2, 2.1)	0.617	0.087
DCCA TAPV (cm/s)	30.0 ± 6.8	30.6 ± 7.7	0.6 (−1.03;2.3)	0.454	26.7 ± 6.1	28.8 ± 6.9	2.1 (0.53;3.65)	0.01	−1.5 (−3.7, 0.764)	0.194	−0.9 (−3.4, 1.6)	0.465	0.023*
DCCA PI	1.54 ± 0.5	1.39 ± 0.4	−0.15 (−0.23; −0.07)	0.001*	1.54 ± 0.4	1.55 ± 1.03	0.013 (−0.3; 0.34)	0.935	−0.16 (−0.49, 0.17)	0.337	−0.06 (−0.42, 0.31)	0.745	0.982
DCCA RI	0.72 ± 0.09	0.68 ± 0.08	−0.04 (−0.05; −0.02)	< 0.001*	0.77 ± 0.33	0.74 ± 0.3	−0.03 (−0.17;0.12)	0.720	−0.01 (−0.15, 0.13)	0.902	−0.01 (−0.17, 0.15)	0.932	0.331

*Note:* Covariates appearing in the model are evaluated at the following values: DCCA PSV = 57.9 cm/s, DCCATAPV = 27.5 cm/s, and ICA PSV = 60.2 cm/s.

Abbreviations: DCCA, distal common carotid artery; EDV, End diastolic velocity; ICA, Internal carotid arteries; MD, mean difference (post minus pre interventional values); *n*, number of vessels (*n* = 42); PI, pulsatility index; PSV (cm/s), peak systolic velocity; RI, resistivity index; TAPV, time averaged peak velocity. *p < 0.05.

^a^ MD, mean difference with no baseline characteristics adjustments.

^b^ MD, mean difference adjusted for baseline characteristics.

The pre and post MCA haemodynamic parameters (PSV, EDV, MFV, RI, and PI) did not differ significantly in both groups (*p* ≥ 0.05), and a small effect size was reported in all parameters (Table S1).

## 4 Discussion

This randomized trial showed that 12 weeks of high‐intensity cycling aerobic exercise training (AET) improved several markers of cerebrovascular structure and vascular function in chronic post‐stroke patients. Compared with the control group, participants who underwent cycling AET demonstrated favorable changes in carotid artery morphology, including reduction in CIMT, and improvements in selected carotid stiffness indices, particularly carotid compliance (CC) and distensibility coefficient (DC). The intervention was however associated with modest improvements in extracranial hemodynamic parameters, whereas no measurable changes were observed in middle cerebral artery (MCA) hemodynamics. Furthermore, and importantly, this study is among the first to examine these effects in post‐stroke patients using advanced multi‐parametric duplex carotid ultrasound and transcranial color‐coded Doppler, and to include novel 3D ultrasound‐based vascular morphological measures. These findings suggest that high‐intensity cycling AET may promote beneficial structural and functional adaptations in the cerebrovascular system after stroke.

### 4.1 Effects of AET on Cerebral Arteries' Morphological and Hemodynamic Features

#### 4.1.1 Cycling AET Effects on CIMT

CIMT is a recognized surrogate indicator for atherosclerosis and an independent predictor of cardiovascular disease risk, highlighting the clinical importance of maintaining lower CIMT values. Although in general, participants in both groups had significantly lower CIMT values post intervention compared to baseline, cycling AET participants exhibited a pronounced reduction in the CIMT with a between group MD of −0.04 (−0.08, 0.001) mm, *p* = 0.043, and an overall medium effect size, Cohen *d* = 0.59. This finding is consistent with prior work showing that AET can reduce CIMT in non‐stroke populations. For example, a previous study reported a significant reduction in CIMT following exercise in healthy adults [34], and similarly, a recent systematic review concluded that an exercise duration lasting > 6 months was associated with a modest reduction in CIMT of 0.02 mm in populations other than chronic post‐stroke patients [35]. However, the magnitude of CIMT improvement observed in our study appears greater compared to that reported in some previous studies [34, 35]. Contrary to our study findings, 3 months of indoor cycling did not significantly alter CIMT in healthy premenopausal women [36]. Differences between studies may be due to the multifactorial nature of adaptation to exercise, with factors such as exercise type, intensity, and individual characteristics influencing the extent of improvement [37]. Although exercise protocols were similar between the two studies, differences in participant characteristics—such as age, gender, and race—may have contributed to observed differences. The previous study included only healthy Caucasian women, while our study involved post‐stroke patients of Asian origin (women and men). In addition, differences in baseline CIMT values may have affected the outcomes, as higher baseline values observed in our study are linked to greater benefits from exercise. Overall, our findings suggest that high‐intensity cycling AET may be particularly relevant for morphological vascular remodeling in the post‐stroke populations.

#### 4.1.2 Cycling AET Effects on Carotid Arterial Stiffness (CAS)

The present study also demonstrated beneficial effects of cycling AET on carotid arterial stiffness. Significant within‐group improvements were observed in the cycling AET group across all evaluated carotid arterial stiffness (CAS) indices (PWV, β‐index, kPa, CC, and DC, all *p* < 0.05). However, only CAS CC and DC showed significant between‐group MDs (all *p* < 0.05), suggesting that the stretching control intervention may not have been physiologically inert. In addition, other factors, such as spontaneous recovery over time and increased general physical activity during the study period, may have contributed to the observed smaller between‐group differences. The between‐group MDs in our study should therefore be interpreted as the added effect of the cycling AET intervention relative to active control rather than to no treatment.

Few studies have examined the effects of cycling AET on carotid arteries' stiffness using these novel ultrasound parameters, and moreover in post‐stroke patients. A study by Bjarnegård et al. [36], concluded that a 3‐month indoor cycling improved carotid artery distensibility in healthy premenopausal women, results which are similar to our current study findings in which significant improvement in carotid compliance was observed in post‐stroke patients (MD = 0.14 mm/kPa, *p* = 0.001). Furthermore, our study reports for the first time noteworthy observations in which 12 weeks of cycling AET significantly reduced the arterial stiffness parameters—CAS (PWV and β‐index) to ranges comparable to those of age‐matched healthy Chinese individuals reported in a recent study [38] (all *p* > 0.05). The remarkable reduction in arterial stiffness following cycling AET points towards a potential future reduction in stroke recurrence as carotid arterial stiffness is an independent factor linked to stroke occurrence, beyond the influence of cardiovascular factors and stiffness of aorta [39, 40]. However, this study did not demonstrate the direct effect of AET on stroke recurrence, an area of future research. The higher standard deviation in MD suggests a significantly varied magnitude of observed changes in arterial stiffness across subjects; hence, further studies are needed to establish possible demographic factors behind such differences.

#### 4.1.3 Effects of Cycling AET on 3D‐Ultrasound Based Carotid Artery Parameters

A notable strength and innovative feature of this study was the use of 3D ultrasound to quantify carotid vascular morphology. Unlike 2D ultrasound, limited to cross‐sectional views, our study utilized 3D that enables precise volumetric quantification of carotid lumen and plaque [41], thus provides more accurate and reliable quantification of vascular changes. Using this approach, our study reported for the first time significant reductions in 3D ultrasound‐based arterial parameters (CLVS (%), CPV, and CVWV) in post‐stroke patients following cycling AET (all *p* < 0.05), and these reductions have significant clinical implications. Since progression of total plaque volume and hemodynamic failure due to plaque build‐up was reported to be predictive of stroke [42], our study findings of reduced CPV and CLVS suggest a possible mitigation against future stroke recurrences. Further studies are recommended to assess the translational benefits of the observed improvements in these novel ultrasound‐based rehabilitation efficacy indicators in mitigating stroke recurrence in post‐stroke patients.

#### 4.1.4 Cycling AET Effects on the Cerebral Arteries' Hemodynamic Parameters

Cycling AET produced modest improvements in extracranial cerebral arteries' hemodynamic parameters, including increases in end‐diastolic velocity and reductions in resistance index and pulsatility index in the distal common carotid artery and internal carotid artery. These changes may reflect improved downstream vascular compliance and reduced vascular resistance, consistent with the structural improvements observed in carotid morphology and stiffness. Contrarily, no changes occurred in MCA hemodynamic parameters (PSV, EDV, MFV, PI, RI). This finding aligns with studies in non‐stroke cohorts, where high‐intensity AET stabilized or reduced cerebral blood flow, possibly to support thermoregulation [43, 44, 45]. Reed et al. [44] reported insignificant increases in cerebral PI despite significant improvements in aortic compliance after 12 weeks of AET in middle‐aged adults, whilst Oliva et al. [43] found no changes in cerebral blood flow following high‐intensity interval treadmill training, although moderate intensity improved PI and RI. Furthermore, Steventon et al. [45] reported moderate intensity cycling AET not to have any effect on cerebrovascular function, in both intracranial or extracranial cerebral arteries (*p* > 0.05). Our findings therefore suggest a well‐functioning cerebral autoregulation mechanism.

### 4.2 Study Strengths and Limitations

To the best of our knowledge, this is the first randomized trial to investigate the effects of high‐intensity cycling AET on detailed cerebrovascular morphology and hemodynamics in chronic post‐stroke survivors using advanced multi‐parametric DCUS, TCCD, and 3D ultrasound techniques. To ensure methodological rigor, all assessments and interventions were performed by trained professionals using validated ultrasonography techniques. Excellent intra‐observer reliability was confirmed for CIMT ICC = 0.99, 95% CI: 0.98–0.995 (ICC (1, k)). Intention‐to‐treat analysis and high adherence (98%) minimized bias from patient exclusions, whereas no injuries were reported, confirming cycling AET a safe and feasible intervention. Post‐intervention assessments were conducted within 10 days (mean: 6 days) to account for possible post‐interventional changes.

Despite strengths, this study is not without limitations. Firstly, medication known to alter blood flow, such as vasodilators, was not controlled. However, their use across the two groups did not significantly differ, reducing the potential for systematic bias related to medication use. In addition, the achieved sample size (*n* = 21) in each group was slightly lower than the a priori target of 24, which may have reduced the power to detect smaller effects for some outcomes. Furthermore, findings may not be fully generalizable to younger post‐stroke populations, as the intervention focused on older stroke survivors. Finally, the lack of long‐term follow‐up prevents conclusions about the sustainability of observed benefits; thus, larger, multicentre future studies with longer follow‐up are recommended.

## 5 Conclusion

This study demonstrated that 12 weeks of high‐intensity cycling AET significantly improved cerebral artery morphology including CIMT, and stiffness parameters (CAS CC and DC) with modest benefits on extracranial cerebral arteries hemodynamics. Future multi‐center studies are recommended to assess long‐term cerebrovascular benefits of cycling AET and potential in mitigating against future stroke recurrences.

## Author Contributions

Conceptualization: S.T.G., M.T.‐C.Y., and M.Y.‐C.P.; methodology: S.T.G., M.T.‐C.Y., M.Y.‐C.P., and X.C.; investigation: S.T.G., J.H.‐Y.Y., V.T.‐K.N., Z.C., and X.H.; data curation: S.T.G., J.H.‐Y.Y., V.T.‐K.N., and J.Q.; formal analysis: S.T.G., Z.C., M.Y.‐C.P., and M.T.‐C.Y.; funding acquisition: M.T.‐C.Y.; project administration: S.T.G. and M.T.‐C.Y.; validation: S.T.G., J.H.‐Y.Y., V.T.‐K.N., Z.C., X.H., J.Q., X.C., M.Y.‐C.P., and M.T.‐C.Y.; visualization: S.T.G., J.H.‐Y.Y., V.T.‐K.N., J.Q., Z.C., X.H., X.C., M.Y.‐C.P., and M.T.‐C.Y.; writing – original draft, S.T.G. and M.T.‐C.Y.; writing – review and editing, S.T.G., J.H.‐Y.Y., V.T.‐K.N., Z.C., J.Q., X.H., X.C., M.Y.‐C.P., and M.T.‐C.Y. All authors have read and approved the final version of the manuscript.

## Funding

This research project was supported by a research studentship grant (R006) and a research project fund (P0035203) of The Hong Kong Polytechnic University, Hung Hom, Kowloon, Hong Kong SAR, China.

## Ethics Statement

The study was approved by the Institutional Review Board (or Ethics Committee) of The Hong Kong Polytechnic University (HSEARS20220714001‐24/08/2022.) and registered under the ClinicalTrials.gov. The study was therefore carried out in accordance with the ethical standards laid down in the 1964 Declaration of Helsinki and its later amendments.

## Consent

All participants provided written informed consent prior to undertaking the study.

## Conflicts of Interest

The authors declare no conflicts of interest.

## Supporting information


**Figure S1:** Image A showing the pre (1) and post (2) estimated marginal means of Carotid intima media thickness (CIMT) (mm) between the cycling AET (red) and stretching (control) (blue) groups.
**Figure S2:** Images showing the pre (1) and post (2) estimated marginal means of the Carotid artery stiffness indices: (A) Pulse wave velocity (CAS PWV), (B) Modulus of elasticity (CAS kPa), (C) Beta Stiffness Index (CAS β), (D) Compliance Coefficient (CAS CC), and (E) Distensibility Coefficient (CAS DC) for the cycling AET (red) and Stretching (control) (blue) groups.
**Figure S3:** Images showing the pre (1) and post (2) estimated marginal means of 3D‐ carotid ultrasound‐based features for the cycling AET (red) and Stretching (control) (blue) groups. (A) Carotid lumen volume stenosis (%) (CLVS), (B) Carotid plaque volume (CPV), (C) Carotid vessel wall volume (CVWV).

**Table S1:** Middle cerebral artery (MCA) hemodynamic features for the cycling AET and Stretching (control) groups and the mean differences (MD).

## Data Availability

The data that support the findings of this study are available on request from the corresponding author. The data are not publicly available due to privacy or ethical restrictions.
