# Supplementary material for: Exercise Effect on Cerebral Artery Hemodynamic and Morphology in Stroke Patients: A Randomized Trial
Source: CNS Neurosci Ther. 2026 May 20;32(5):e70942. doi: 10.1002/cns.70942 (PMC13239555; doi:10.1002/cns.70942)
Supplement: Supplementary file 1 — Figure S1: Image A showing the pre (1) and post (2) estimated marginal means of Carotid intima media thickness (CIMT) (mm) between the cycling AET (red) and stretching (control) (blue) groups. Figure S2: Images showing the pre (1) and post (2) estimated marginal means of the Carotid artery stiffness indices: (A) Pulse wave velocity (CAS PWV), (B) Modulus of elasticity (CAS kPa), (C) Beta Stiffness Index (CAS β), (D) Compliance Coefficient (CAS CC), and (E) Distensibility Coefficient (CAS DC) for the cycling AET (red) and Stretching (control) (blue) groups. Figure S3: Images showing the pre (1) and post (2) estimated marginal means of 3D‐ carotid ultrasound‐based features for the cycling AET (red) and Stretching (control) (blue) groups. (A) Carotid lumen volume stenosis (%) (CLVS), (B) Carotid plaque volume (CPV), (C) Carotid vessel wall volume (CVWV). [file CNS-32-e70942-s002.docx]

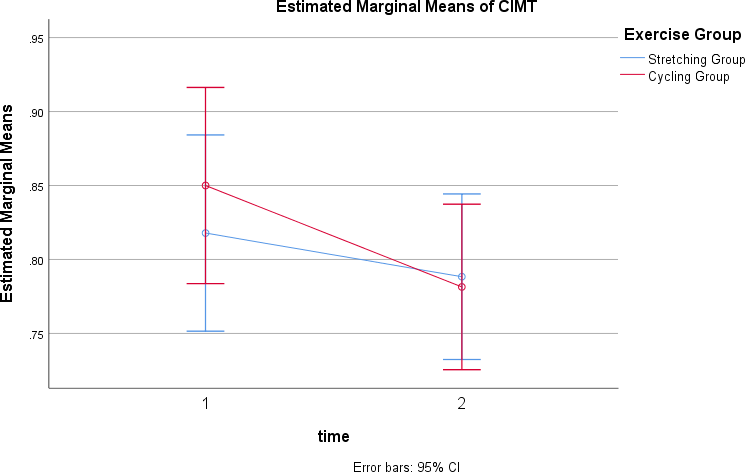


**A**

**Figure S1:** Image A showing the pre (1) and post (2) estimated marginal means of Carotid intima media thickness (CIMT) (mm) between the cycling AET (red) and stretching (control) (blue) groups.


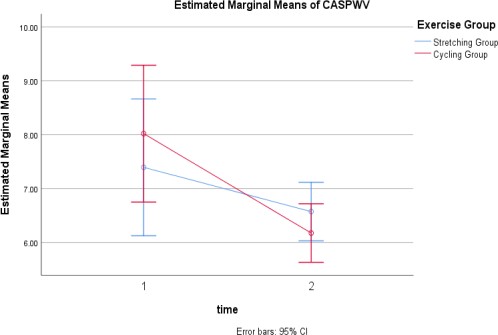

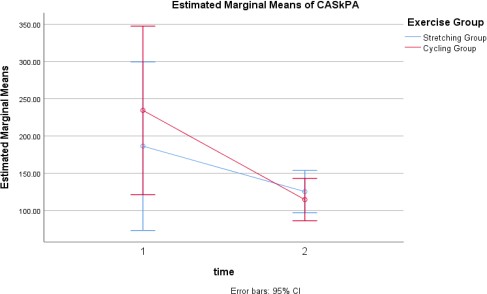

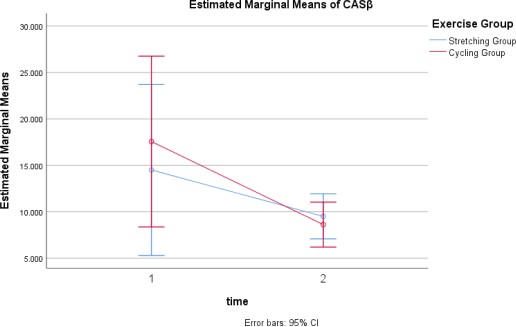

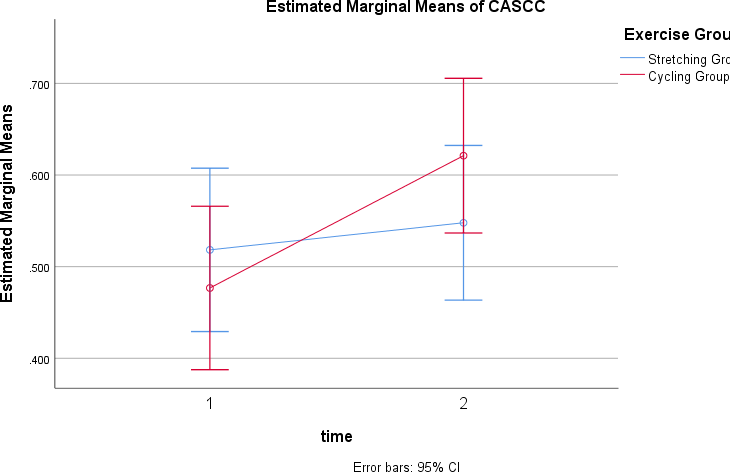

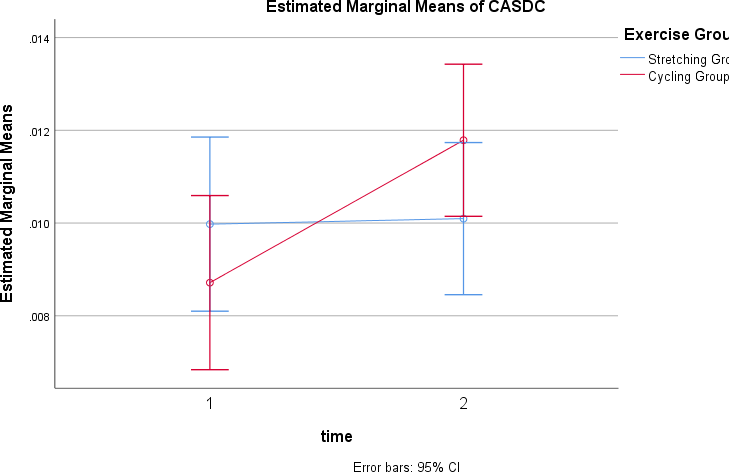


**A**

**B**

**C**

**D**

**E**

**Figure S2**: Images showing the pre (1) and post (2) estimated marginal means of the Carotid artery stiffness indices: A. Pulse wave velocity (CAS PWV), B. Modulus of elasticity (CAS kPa), C. Beta Stiffness Index (CAS β), D. Compliance Coefficient (CAS CC), and E. Distensibility Coefficient (CAS DC) for the cycling AET (red) and Stretching (control) (blue) groups.


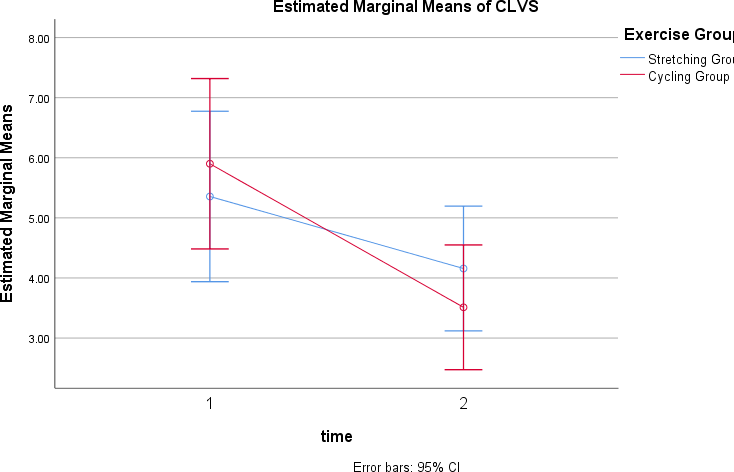

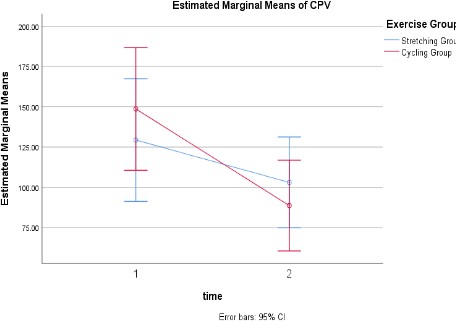

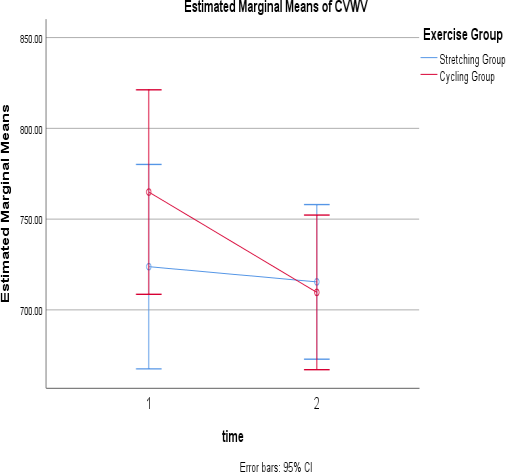


**A**

**B**

**C**

**Figure S3**: Images showing the pre (1) and post (2) estimated marginal means of 3D- carotid ultrasound-based features for the cycling AET (red) and Stretching (control) (blue) groups. A. Carotid lumen volume stenosis (%) (CLVS), B. Carotid plaque volume (CPV), C. Carotid vessel wall volume (CVWV)
