# Supplementary material for: Exercise Effect on Cerebral Artery Hemodynamic and Morphology in Stroke Patients: A Randomized Trial
Source: CNS Neurosci Ther. 2026 May 20;32(5):e70942. doi: 10.1002/cns.70942 (PMC13239555; doi:10.1002/cns.70942)
Supplement: Supplementary file 2 — Table S1: Middle cerebral artery (MCA) hemodynamic features for the cycling AET and Stretching (control) groups and the mean differences (MD). [file CNS-32-e70942-s001.docx]

| **MCA Hemodynamic Parameter** | **Groups** | | | | | | | | **Between Group Comparisons** | | | | |
| --- | --- | --- | --- | --- | --- | --- | --- | --- | --- | --- | --- | --- | --- |
|  | **Cycling AET— Interventional** | | | | **Stretching— Control** | | | | **Btwn Group MD**—**no baseline adjustments** | | **Btwn Group**  **^*^MD**—**baseline adjustments** | |  |
|  | Pre-Cycle | Post-Cycle | Cycle MD, 95% CI | p-values | Pre-Stretch | Post-Stretch | Stretch MD,95% CI | p-values | MD, 95% CI | p-values | ^*^MD, 95% CI | p-values |  |
| PSV  (cm/s) | 81.4 ± 26.5 | 78.2±24.7 | -3.2 (-7.43;1.04) | 0.133 | 78.8±32.8 | 75.0±32.1 | -3.8(-8.7; -1.6) | 0.120 | 0.63(-5.75;7.0) | 0.845 | 0.86 (-5.57, 7.28) | 0.790 |  |
| EDV (cm/s) | 31.5± 13.3 | 30.4±13.8 | -1.15(-4.1;1.79) | 0.432 | 26.5±16.4 | 24.7±14.2 | -1.8(-6.81;3.18) | 0.459 | 0.67(-4.63;6.0) | 0.802 | 1.04 (-4.47, 6.55) | 0.705 |  |
| MFV  (cm/s) | 51.5±17.7 | 48.3±16.6 | -3.23(-6.5;0.004) | 0.05 | 47.8±20.4 | 47.4±20.7 | -0.41(-3.8;3.0) | 0.804 | -2.82(-7.5;1.9) | 0.234 | -2.5 (-7.32,2.39) | 0.312 |  |
| RI | 0.61±0.12 | 0.62±0.14 | 0.003(-0.03,0.04) | 0.868 | 0.67±0.19 | 0.66±0.19 | -0.01(-0.06;0.03) | 0.555 | 0.02(-0.04; 0.07) | 0.545 | 0.01 (-0.05, 0.07) | 0.770 |  |
| PI | 1.00±0.27 | 1.06±0.48 | 0.067(-0.6;0.20) | 0.303 | 1.18±0.56 | 1.09±0.47 | -0.09(-0.21;0.04) | 0.179 | 0.15(0.09; -0.03) | 0.105 | 0.12 (-0.08, 0.32) | 0.225 |  |

**Table S1 Middle cerebral artery (MCA) hemodynamic features for the cycling AET and Stretching (control) groups and the mean differences (MD)**

Abbreviations: PSV (cm/s) —Peak systolic velocity, EDV—End diastolic velocity, MFV—mean flow velocity, PI—pulsatility index, RI—resistivity index, M.D—mean difference (post minus pre interventional values), *MD—mean difference adjusted for baseline characteristics.
